# Supplementary material for: Effects of Through-Bond and Through-Space Conjugations on the Photoluminescence of Small Aromatic and Aliphatic Aldimines
Source: Molecules. 2022 Nov 19;27(22):8046. doi: 10.3390/molecules27228046 (PMC9693914; doi:10.3390/molecules27228046)
Supplement: Supplementary file 1 [file molecules-27-08046-s001.zip › molecules-1995199-supplementary.pdf]

Supplementary Materials

Effects of Through-Bond and Through-Space Conjugations on the Photoluminescence of Small Aromatic and Aliphatic Aldimines

Peifeng Zhuang [1], Chang Yuan [2], Yunhao Bai [1], Changcheng He [1,*], Jiayu Long [1], Hongwei Tan [2,*] and Huiliang Wang [1,*]

[1] Beijing Key Laboratory of Energy Conversion and Storage Materials, College of Chemistry, Beijing Normal University, Beijing 100875, China

[2] Key Laboratory of Theoretical and Computational Photochemistry, Ministry of Education, College of Chemistry, Beijing Normal University, Beijing 100875, China

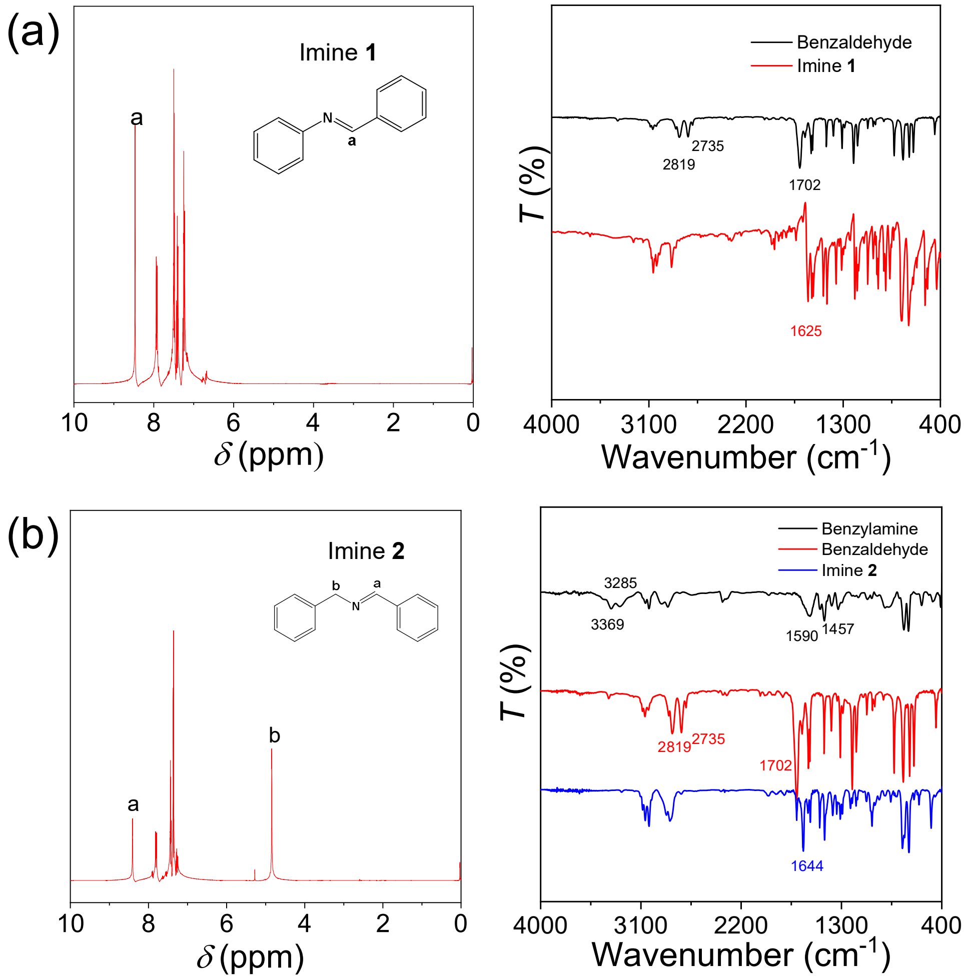

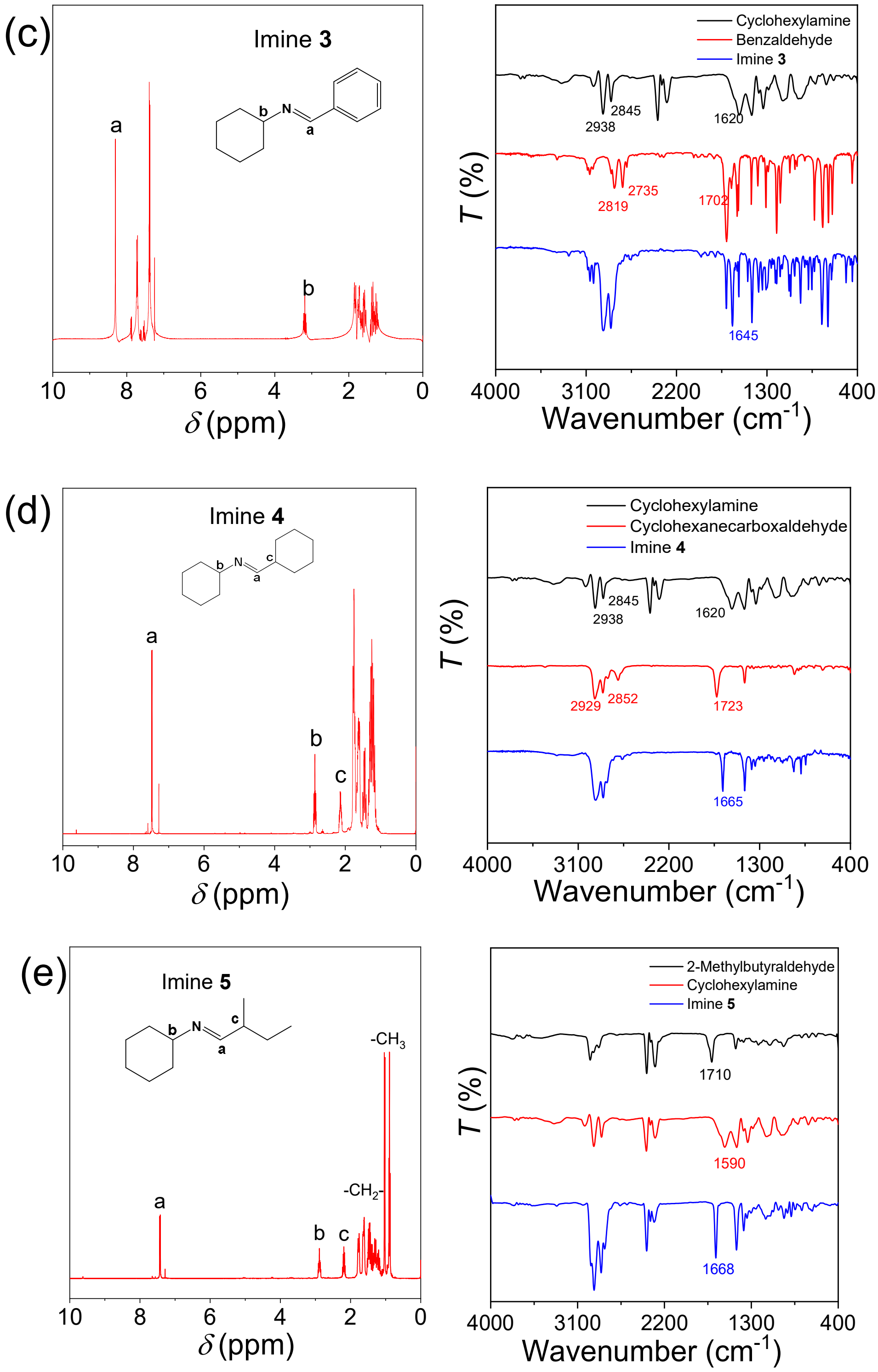

(c)
Imine 3
a
b
N
10 8 6 4 2 0
δ (ppm)
Cyclohexylamine
Benzaldehyde
Imine 3
2845
2938
1620
2735
2819
1702
1645
T (%)
4000 3100 2200 1300 400
Wavenumber (cm-1)
(d)
Imine 4
a
b
c
N
10 8 6 4 2 0
δ (ppm)
Cyclohexylamine
Cyclohexanecarboxaldehyde
Imine 4
2845
2938
1620
2929
2852
1723
1665
T (%)
4000 3100 2200 1300 400
Wavenumber (cm-1)
(e)
Imine 5
a
b
c
N
-CH3
-CH2-
10 8 6 4 2 0
δ (ppm)
2-Methylbutyraldehyde
Cyclohexylamine
Imine 5
1710
1590
1668
T (%)
4000 3100 2200 1300 400
Wavenumber (cm-1)

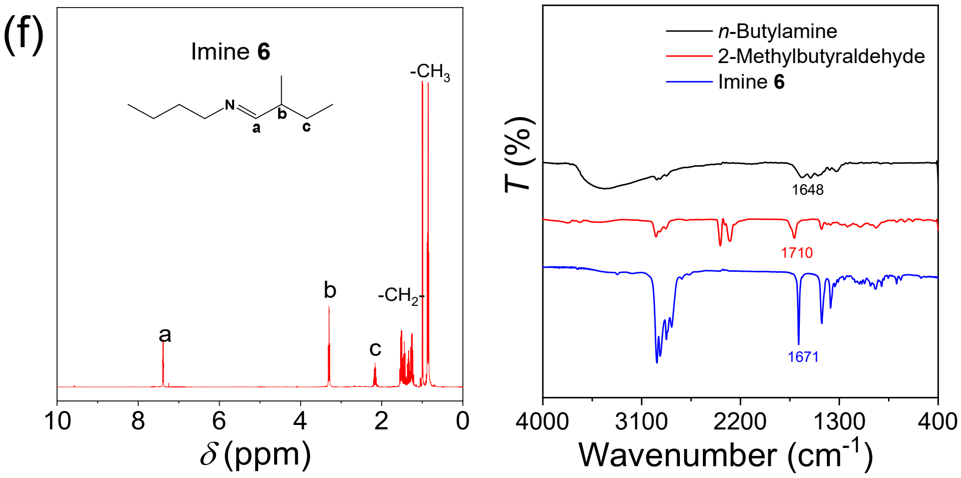

Figure S1. ^{1}H NMR (left) spectra of the imines **1-6,** and FT-IR (right) spectra of the imines with comparison to the corresponding primary amines and aldehydes.

Table S1 Characterization results of imines **1-6**.

Chemical formula	¹H NMR<br>δ (ppm)	FT-IR<br>ν (cm^{-1})	m/z
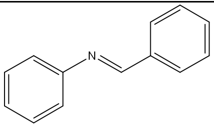<br>**Imine 1**	8.47 (s, 1H), 8.03-7.78 (2H), 7.75 (3H), 7.41 (4H), 7.23 (1H)	3057/3013, 2891/2854, 1625	181.09
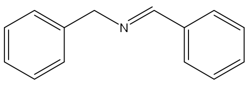<br>**Imine 2**	8.41 (t, 1H), 7.81 (2H), 7.44 (3H), 7.35 (5H), 4.84 (2H)	3085/3060/3029, 2873/2835, 1644	195.11
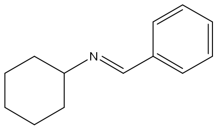<br>**Imine 3**	8.31 (s, 1H), 7.73 (2H), 7.38 (3H), 3.19 (m, 1H), 1.99-1.15 (10H)	3075/3017, 2931/2851, 1645	187.14
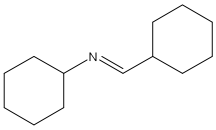<br>**Imine 4**	7.44 (d, 1H), 2.82 (m, 1H), 2.10 (1H), 1.89-0.92 (20H)	2939/2852/2812, 1665, 1442	193.19
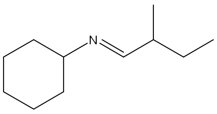<br>**Imine 5**	7.38 (d, 1H), 2.85 (m, 1H), 2.16 (m, 1H), 1.82-1.11 (12H), 0.98 (d, 3H), 0.86 (t, 3H)	2960/2929/2858/2821, 1668, 1453	167.18
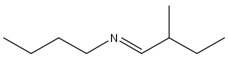<br>**Imine 6**	7.40 (m, 1H), 3.30 (m, 2H), 2.18 (m, 1H), 1.61-1.16 (6H), 0.99 (d, 3H), 0.87 (t, 3H), 0.84 (t, 3H)	2962/2924/2872/2827, 1671, 1460	141.16

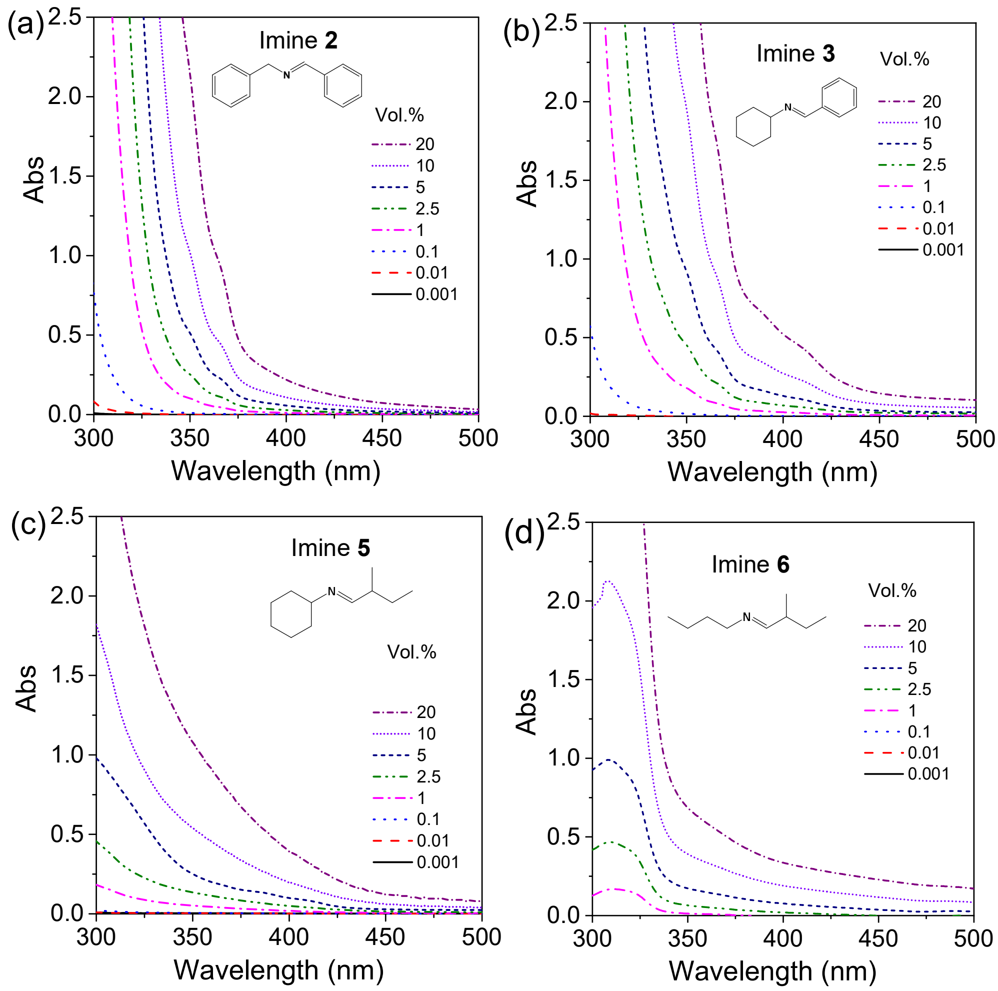

Figure S2. UV-vis absorption spectra of imines **2** (a), **3** (b), **5** (c) and **6** (d) ethanol solutions with different concentrations.

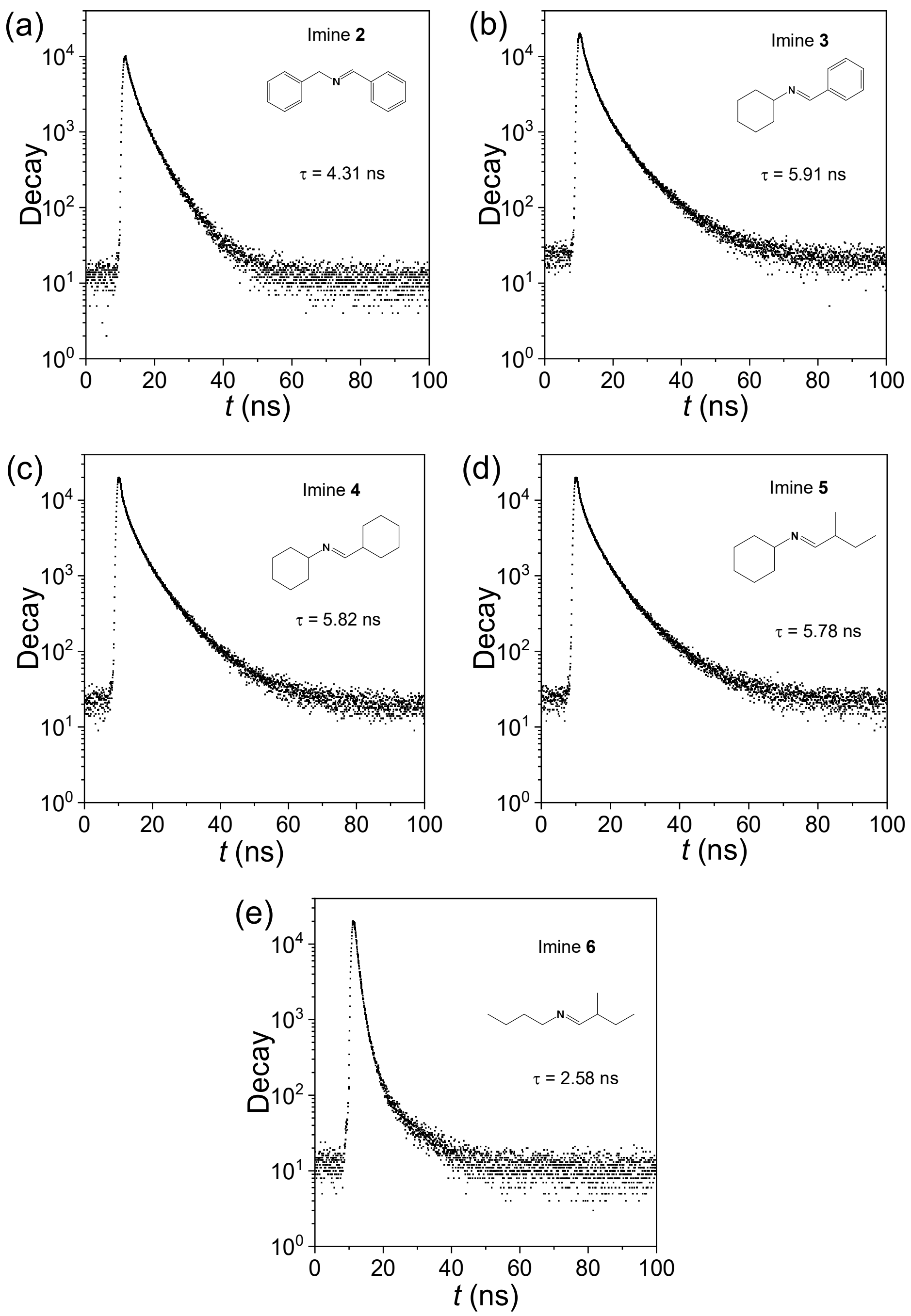

Figure S3. Lifetimes of imines **2**-**6** ethanol solutions (5 vol.%).

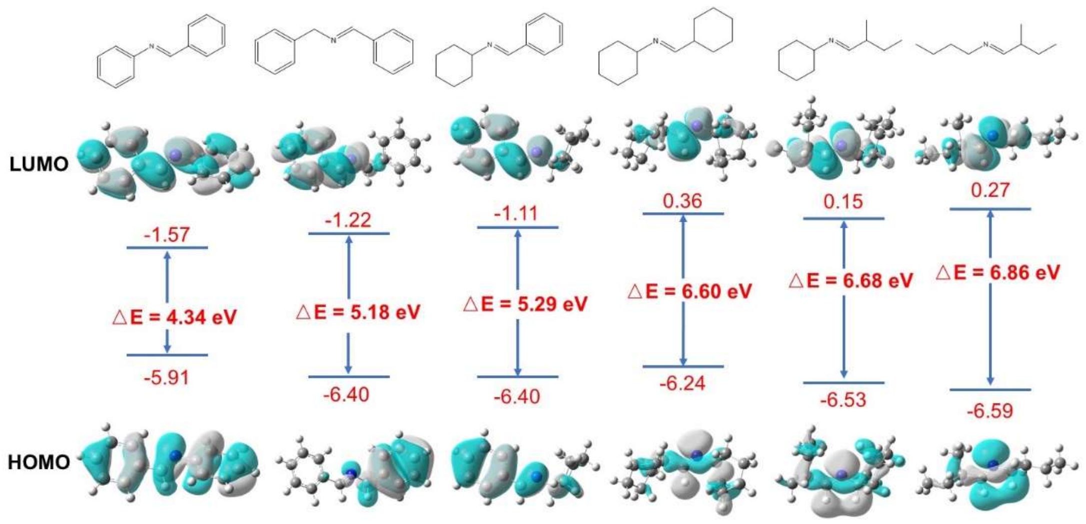

Figure S4. HUMO and LUMO orbitals of different imines in *trans*-configuration.

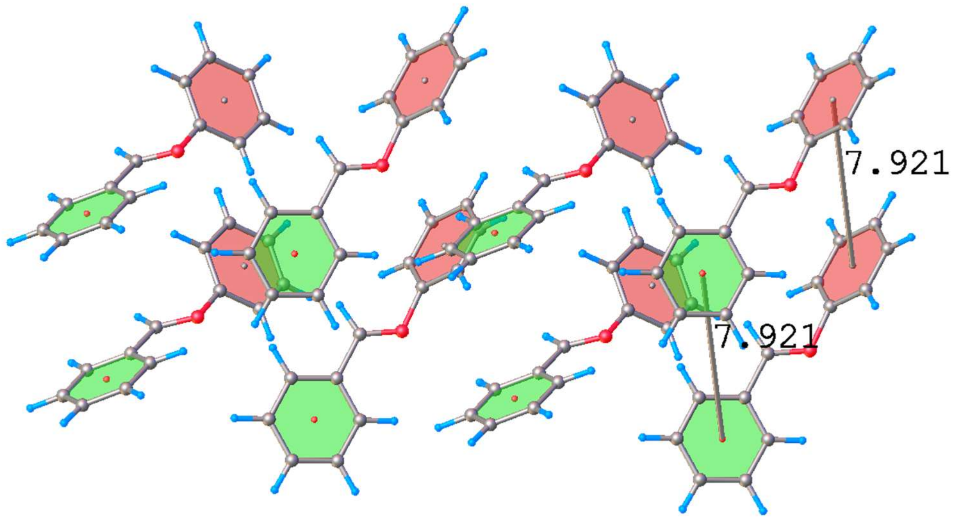

Figure S5. Molecular arrangement in the crystal of imine **1**.

Table S2. Energies of imines **1-6** in the *cis* and *trans* forms and their energy differences.

Form	Energy (kCal/mol)					
	Imine **1**	Imine **2**	Imine **3**	Imine **4**	Imine **5**	Imine **6**
cis	-349478.97	-374152.24	-351749.44	-354028.65	-305437.77	-256838.45
trans	-349483.62	-374159.02	-351766.92	-354033.20	-305446.29	-256842.41
ΔE	4.66	6.796	17.48	4.54	8.53	3.97

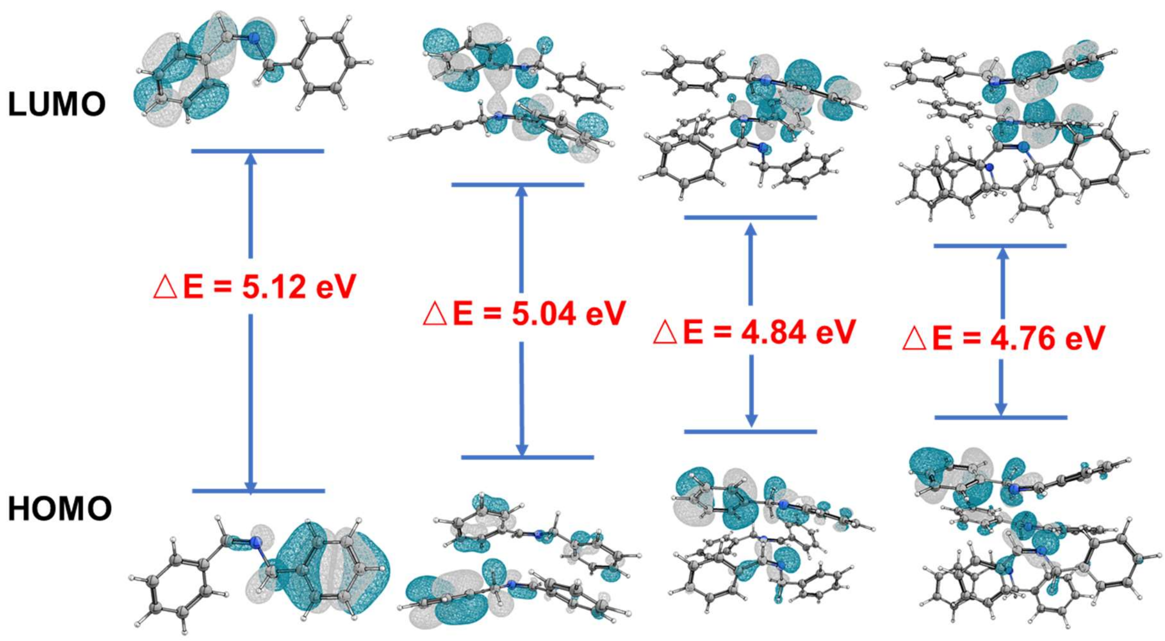

Figure S6. Optimized conformations of various imine **2** molecule(s) and their molecular orbital surfaces of HOMOs and LUMOs. From left to right, the molecule number in the model is 1 to 4.

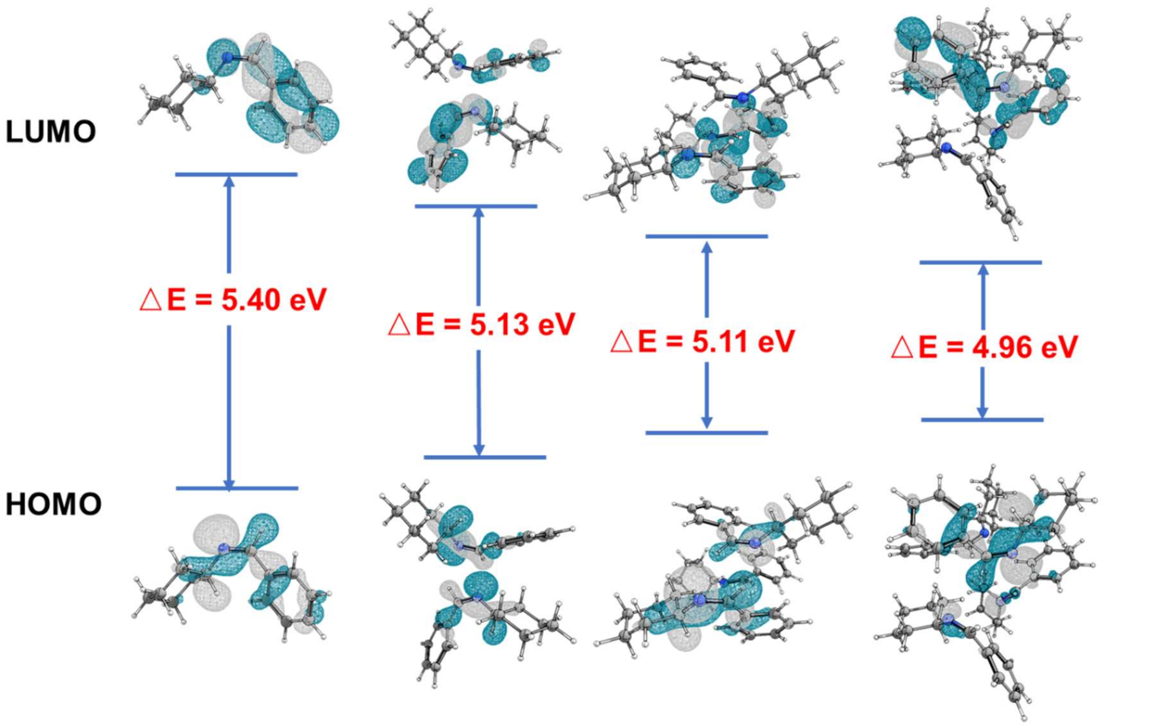

Figure S7. Optimized conformations of various imine **3** molecule(s) and their molecular orbital surfaces of HOMOs and LUMOs. From left to right, the molecule

number in the model is 1 to 4.

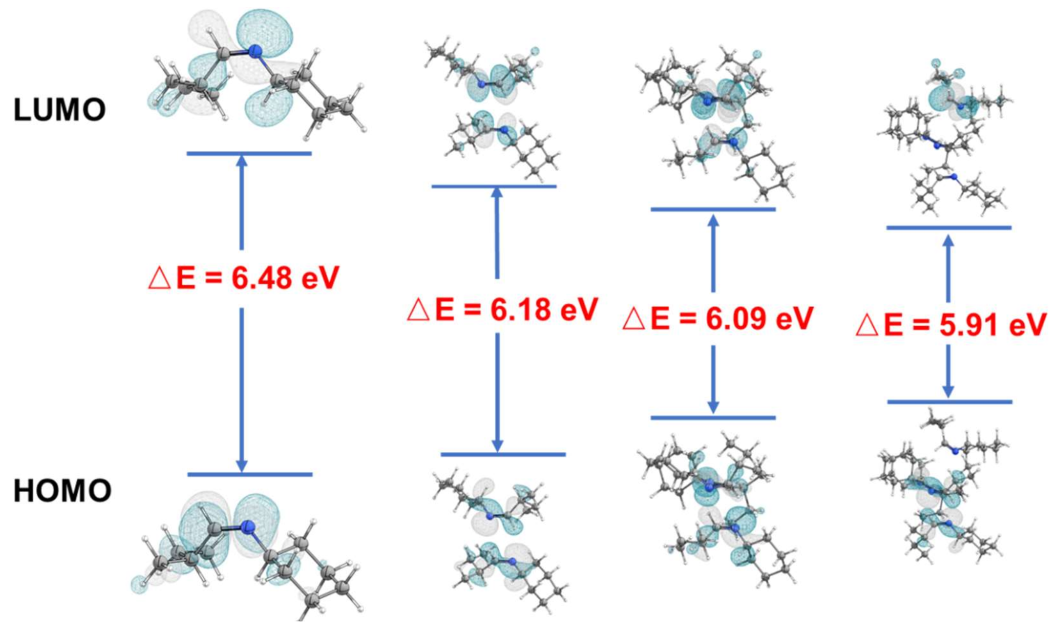

Figure S8. Optimized conformations of various imine **5** molecule(s) linked together by hydrogen bonds and their molecular orbital surfaces of HOMOs and LUMOs. From left to right, the molecule number in the model is 1 to 4.

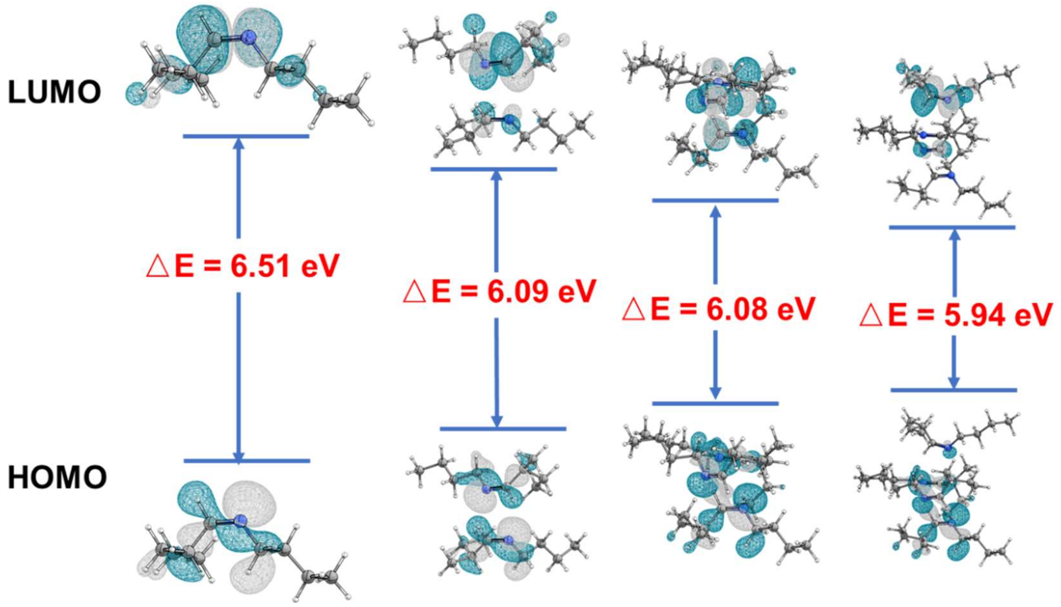

Figure S9. Optimized conformations of various imine **6** molecule(s) linked together by hydrogen bonds and their molecular orbital surfaces of HOMOs and LUMOs. From left to right, the molecule number in the model is 1 to 4.
